# A Comparison of Stage Conversion in the Coccidian Apicomplexans *Toxoplasma gondii*, *Hammondia hammondi*, and *Neospora caninum*

**DOI:** 10.3389/fcimb.2020.608283

**Published:** 2020-12-03

**Authors:** Sarah L. Sokol-Borrelli, Rachel S. Coombs, Jon P. Boyle

**Affiliations:** University of Pittsburgh, Department of Biological Sciences, Kenneth P. Dietrich School of Arts and Sciences, Pittsburgh, PA, United States

**Keywords:** *Toxoplasma gondii*, *Hammondia hammondi*, *Neospora caninum*, bradyzoite, tissue cysts

## Abstract

Stage conversion is a critical life cycle feature for several Apicomplexan parasites as the ability to switch between life forms is critical for replication, dissemination, pathogenesis and ultimately, transmission to a new host. In order for these developmental transitions to occur, the parasite must first sense changes in their environment, such as the presence of stressors or other environmental signals, and then respond to these signals by initiating global alterations in gene expression. As our understanding of the genetic components required for stage conversion continues to broaden, we can better understand the conserved mechanisms for this process and unique components and their contribution to pathogenesis by comparing stage conversion in multiple closely related species. In this review, we will discuss what is currently known about the mechanisms driving stage conversion in *Toxoplasma gondii* and its closest relatives *Hammondia hammondi* and *Neospora caninum*. Work by us and others has shown that these species have some important differences in the way that they (1) progress through their life cycle and (2) respond to stage conversion initiating stressors. To provide a specific example of species-specific complexities associated with stage conversion, we will discuss our recent published and unpublished work comparing stress responses in *T. gondii* and *H. hammondi*.

## Introduction

Eukaryotic parasites with multi-host life cycles have the unique challenge of persisting in a variety of different environments. Parasites of this nature must exist in a life form that is permissible and optimized for growth, survival, and transmission in a given host. The ability to adopt these specific life forms is especially critical for tissue cyst forming coccidia in the Sarcocystidae family which includes *Sarcocystis, Frenkelia, Besnoitia, Cystoisopora, Toxoplasma, Hammondia, and Neospora* species (Smith, 1981; Duszynski et al., 2018). Here, we will discuss the current knowledge about how three closely related parasites from this family, *Toxoplasma gondii*, *Hammondia hammondi*, and *Neospora caninum*, approach the challenges associated with multi-host life cycles. These three mammalian parasites follow heteroxenous, two host life cycles (Frenkel et al., 1970; Frenkel and Dubey, 1975; McAllister et al., 1998; Dubey, 2009; Dubey and Ferguson, 2015) where they must survive in a variety of cell types with varying immune pressures. In order to be successful in their hosts, these parasites are capable of converting to life forms that each serve an important function for survival and fitness. Although these parasites exhibit the same infectious life forms, they have fundamental differences in their life cycles and stage conversion strategies. This review will focus on the critical differences in stage conversion between tachyzoites, the rapidly replicating life form, and bradyzoites, the slower growing parasites comprising tissue cysts, in *T. gondii, H. hammondi*, and *N. caninum*.

**Figure 1:**
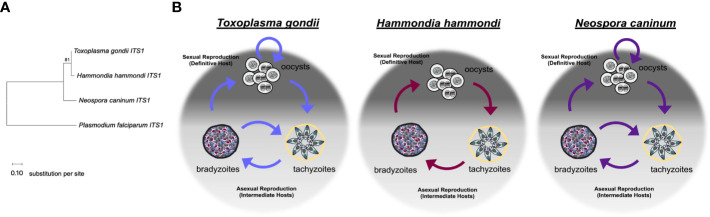
Relationships between *Toxoplasma gondii*, *Hammondia hammondi*, and *Neospora caninum*. **(A)** Neighbor-joining tree depicting relationship between the ITS1 (internal transcribed spacer 1) sequences of *T. gondii*, *H. hammondi*, and *N. caninum*. *P. falciparum* is used as an outgroup. ITS1 sequences were chosen to highlight the relationship between these parasite species due to high variability often attributed to these non-coding sequences. Sequences were obtained from GenBank. Bootstrap values for 1000 replicates are indicted. Scale bar represents the substitutions per site. **(B)** Diagrams showing the life cycle of *T. gondii*, *H. hammondi*, and *N. caninum*.

*T. gondii*, *H. hammondi*, and *N. caninum* are all closely related (**Figure 1A**), obligate intracellular parasites that follow two-host life cycles (See detailed description in section 2). These parasites species also share the same class of organelles and have several shared genomic features. *T. gondii* and *H. hammondi* share >95% of their genomes in near perfect synteny (Walzer et al., 2013), while *T. gondii* and *Neospora caninum* genomes are >81% syntenic (Adomako-Ankomah et al., 2014). Conservative predictions have identified 7,095 shared orthologs between *H. hammondi* and *T. gondii* and 6,308 orthologs between *T. gondii* and *N. caninum* (Lorenzi et al., 2016). Despite these genetic similarities, they exhibit substantial differences in pathogenesis, host range, and life cycles.

*Toxoplasma gondii* has a broad host range and can infect all warm-blooded animals including birds (Tenter et al., 2000). *T. gondii* is responsible for the human disease toxoplasmosis and has infected approximately 2 billion people worldwide (Furtado et al., 2011). Although infections are often asymptomatic, *T. gondii* infections persist in the host in the form of tissue cysts, the life stage that contributes to chronic infection (Remington and Cavanaugh, 1965). Tissue cysts cannot be cleared by the host immune response nor can they be eliminated by known antiparasitic drugs (Tomavo and Boothroyd, 1995; Sullivan and Jeffers, 2012). *T. gondii* infections are thought to be long term, as bradyzoite containing tissue cysts reside in host tissue for extended periods of time and can reactivate causing clinical disease (Tenter et al., 2000; Weiss and Kim, 2000; Rougier et al., 2017). *T. gondii* tissue cysts can cause severe complications for immunocompromised individuals, such as HIV/AIDS and organ transplant patients, when latent infections reactivate into highly replicative life forms and result in tissue damage (Derouin et al., 1987; Derouin et al., 2008). Furthermore, bradyzoite containing tissue cysts maintained in the animal population contributes to the spread of *T. gondii* to both animals and humans, as *T. gondii* is the leading cause of death caused by a food borne illness in the United States (CDC - Toxoplasmosis; Tenter et al., 2000; Tenter, 2009). Individuals, even those who are immunocompetent, infected with *T. gondii* can also develop ocular toxoplasmosis. *T. gondii* is capable of invading and replicating in the retina which can result in severe retinal damage that can lead to blindness (Ozgonul and Besirli, 2017).

Despite being the nearest living relative of *T. gondii*, *H. hammondi* is avirulent in comparison to *T. gondii*. *H. hammondi* is not known to infect humans and is not known to cause natural disease in any animal model (Dubey and Sreekumar, 2003). *H. hammondi* has a restricted host range when compared to *T. gondii*, as its only known natural intermediate hosts are rodents (Mason, 1978), roe deer (Entzeroth and Scholtyseck, 1978), and goats (Shimura and Ito, 1987). Despite having a limited natural host range, *H. hammondi* has been shown to experimentally infect monkeys (Dubey and Wong, 1978), dogs (Dubey, 1975), rabbits, and pigs (Dubey and Sreekumar, 2003), yet is unable to infect birds (Wallace, 1975; Dubey and Streitel, 1976). Despite this slight expansion in experimental host range, nonmurine animals are considered poor hosts for *H. hammondi* as infections do not result in robust chronic infection as *H. hammondi*-infected tissues from these animals produce fewer oocysts compared to *H. hammondi*-infected tissues from murine hosts upon sexual reproduction (Dubey and Streitel, 1976).

In stark contrast to the avirulent nature of *H. hammondi*, *N. caninum* is the major cause of bovine abortion, resulting in losses of over a billion dollars worldwide in cattle industries (Dubey et al., 2007; Goodswen et al., 2013). Like with *T. gondii*, the formation of tissue cysts is also critical for the transmission and survival of *N. caninum*. The reactivation of bradyzoite-containing tissue cysts into fast replicating tachyzoites during bovine pregnancy results in efficient transmission to the fetus. Efficient transmission, persistence, and recrudescence in asymptomatic cows is a significant biotic constraint in these agriculturally important animals, and currently there is no treatment or vaccine for bovine neosporosis (Buxton et al., 2002; Dubey et al., 2006; Williams and Trees, 2006; González-Warleta et al., 2018). While cattle are considered the major intermediate host, *N. caninum* can also infect a number of other domestic and wild ruminant species where its infection can result in disease (Dubey et al., 2013).

## Life Cycles of *Toxoplasma gondii, Hammondia hammondi*, and *Neospora caninum*

*Toxoplasma gondii* follows a facultative homoxenous/heteroxenous two host life cycle (**Figure 1B**). Sexual reproduction of *T. gondii* occurs in its definitive hosts which include members of the *Felidae* genus and produces millions of orally infectious oocysts that are environmentally stable. Each oocyst contains 8 sporozoites contained within 2 sporocysts that are surrounded by an oocyst wall (Frenkel et al., 1970; Dubey, 1998; Dubey, 2009). When oocysts are ingested by an intermediate host, the sporozoites will excyst within the host digestive system and invade the intestinal epithelium. Once in the intestinal epithelium, sporozoites differentiate into tachyzoites, which are the fast growing life stage responsible for acute infection. During a primary infection of a naïve intermediate host, *T. gondii* tachyzoites are capable of crossing the placenta, which can result in vertical transmission of this parasite (McAuley, 2014). Eventually, *T. gondii* tachyzoites differentiate into bradyzoites, the slow growing life stage associated with chronic infection. Bradyzoites are found within tissue cysts which typically reside in the central nervous system or in skeletal or cardiac muscle (Dubey et al., 1998). Bradyzoite tissue cysts are orally infectious to the definitive host. When ingested by a definitive host, tissue cysts will differentiate into the sexual stages resulting in the production of millions of oocysts, thus completing the life cycle (Dubey and Frenkel, 1972). Additionally, cats have been described as complete hosts for *T. gondii* because they support proliferation of tachyzoites and bradyzoites in extra-intestinal tissues in addition to supporting sexual life stages and sexual reproduction in intestinal tissues (Frenkel, 1977). One of the most critical components of the *T. gondii* life cycle is reactivation. Reactivation can occur when 1) a chronically infected host becomes immunocompromised which results in rapid proliferation and dissemination of tachyzoites or 2) when a naïve intermediate host ingests *T. gondii* tissue cysts, the bradyzoites contained within these tissue cysts are released during digestion and differentiate back to tachyzoites, which then disseminate and cause acute infection in the host before encysting during chronic infection. Reactivation results in expanded transmission for *T. gondii*, as it allows for the parasites to be continually propagated through asexual reproduction in intermediate hosts and underlies disease progression in immunocompromised hosts (Dubey et al., 1998). To date, the molecular determinants of this unique ability have not been elucidated, but it has likely had a dramatic impact on the global distribution and broad host range of *T. gondii*.

In comparison to *T. gondii*, *Hammondia hammondi* follows a strict obligate heteroxenous life cycle (**Figure 1B**). Sexual reproduction only occurs within the intestinal epithelium of the definitive host, which, like *T. gondii*, includes felids. *H. hammondi* oocysts, like *T. gondii* oocysts, are orally infectious to intermediate hosts (Frenkel and Dubey, 1975; Dubey and Ferguson, 2015). Unlike *T. gondii* infections, feline hosts can only support *H. hammondi* enteroepithelial stages and do not support extraintestinal infections (Dubey and Sreekumar, 2003). Furthermore, during asexual reproduction *H. hammondi* remain infectious to intermediate hosts until they terminally differentiate into tissue cyst stages (Dubey and Sreekumar, 2003; Sokol et al., 2018) that are only orally infectious to definitive feline hosts. It is hypothesized that terminally differentiated *H. hammondi* parasites are incapable of reactivation, however, this has yet to be tested in head-to-head comparisons with *T. gondii*. Furthermore, *H. hammondi* is not known to be capable of vertical transmission (Dubey and Streitel, 1976).

Like *T. gondii, N. caninum* also follows a complex facultative heteroxenous life cycle (**Figure 1B**). As described for *T. gondii* and *H. hammondi*, sexual reproduction for *N. caninum* infections in the definitive host (in this case canines rather than felines) results in oocyst production and subsequent excretion in host feces. *N. caninum* oocysts are environmentally stable and contain sporozoites that are orally infectious to intermediate hosts. *N. caninum* undergoes sexual reproduction in members of the *Canis* genus (Donahoe et al., 2015) and it is presumed that sexual reproduction occurs in enteroepithelial cells similar to *T. gondii* and *H. hammondi* in felid infections, however, little is known about the sexual life cycle of *N. caninum* infections. The first report of enteroepithelial developmental stages of *N. caninum* was published in 2015 and identified oocyst and schizont-like structures in epithelia in a naturally infected dog (Kul et al., 2015) *N. caninum* tachyzoites and bradyzoites can be found in both intermediate and definitive host tissue, primarily in the central nervous system (Dubey et al., 2002; Dubey et al., 2007). *N. caninum* infections in canids are similar to *T. gondii* in felids in that dogs can be considered complete hosts for *N. caninum* and support replication of all three infectious life stages. As with *T. gondii*, transmission via carnivory of infectious tissue cysts is important for horizontal transmission of *N. caninum* to the definitive host. Although experimental infections demonstrate *N. caninum* tissue cysts are orally infectious (Lindsay and Dubey, 1990; Lindsay et al., 2001), the ingestion of sporulated oocysts is the only known natural mode of horizontal transmission to cattle (De Marez et al., 1999; Kano et al., 2005; Eastick and Elsheikha, 2010). Vertical transmission is the most frequent mode of transmission in bovine hosts and both endogenous (reactivated) and exogenous (primary) transplacental infections occur (Williams et al., 2009). During vertical transmission, *N. caninum* converts into fast-replicating tachyzoites which cross the placenta to transmit to offspring often resulting in abortion. Following transmission, *N. caninum* converts to bradyzoites to evade the host immune responses and tissue cysts form. It is thought the interconversion from bradyzoite to tachyzoite, allows for one infected animal to transmit the parasite to offspring repeatedly. In support of this, recrudescence and transmission have been described in cattle (Williams et al., 2009), sheep (Gutiérrez-Expósito et al., 2020) and in dogs (Cole et al., 1995; Barber and Trees, 1998; Heckeroth and Tenter, 2007). As for *T. gondii*, the means by which *N. caninum* is capable of moving both forwards and backwards in its life cycle is unknown.

Regardless of parasite species, the formation of bradyzoites is a critical step in the transmission of *T. gondii*, *H. hammondi*, and *N. caninum*, despite clear differences in their life cycles. Bradyzoites are necessary for transmission as their cyst wall provides protection during passage through the mammalian gut (Jacobs et al., 1960), and they are also required for sexual reproduction which dramatically expands the number of infectious oocysts available to infect new hosts. Even though the definitive hosts for *T. gondii* and *N. caninum* (felines and canines, respectively), are complete hosts (host that can support both sexual and asexual replication), bradyzoite formation within these animals is still needed in order for sexual reproduction to occur. Bradyzoites also allow the parasites to remain in a host for extended period of time as they cannot be cleared by the host immune system. Finally, interconversion that occurs in *T. gondii* and *N. caninum* allows for these parasites to be transmitted completely independently of the definitive host, a remarkable trait given the vastly different species that can serve as intermediate hosts for these parasites. For these reasons, understanding the mechanisms driving the conversion of tachyzoites to bradyzoites and how it compares between species is necessary to manage the many manifestations of disease caused by these organisms. Moreover, by identifying the developmental sensors, triggers, and components in these species and comparing their activities in each may lead to a better understanding of how this process is regulated on the molecular level.

## Inducers of Bradyzoite Development

While environmental signals are likely at the heart of stage conversion in *T. gondii* and its near relatives, the precise mechanisms used to respond to these signals have remained elusive. However, the effect of various signals on inducing life stage development, specifically tissue cyst formation, has been well studied in *T. gondii*. Furthermore, recent work has begun to identify distinct responses to these triggers in *H. hammondi*, indicating the presence of a divergent stress response in this species.

### Toxoplasma gondii

Bradyzoite formation in *T. gondii* has been extensively studied. An important, but underappreciated, fact is that most strains of *T. gondii* are capable of spontaneously converting into bradyzoites when grown in vitro in a variety of different host cells (Lindsay et al., 1991; Lindsay et al., 1993). When *T. gondii* infections are initiated with parasites derived from sporozoites or from tachyzoites or bradyzoites that have not be extensively passaged following isolation, the parasites will first grow as tachyzoites but will later form tissue cysts that express bradyzoite markers and/or are able to induce oocyst shedding when fed to cats (Lindsay et al., 1991; Jerome et al., 1998). This phenomenon of spontaneous stage conversion in *T. gondii* was most thoroughly studied in *T. gondii* strain VEG. When infections are initiated with *T. gondii* VEG sporozoites, the parasites differentiate into a rapidly growing tachyzoite stage resembling many lab-adapted strains like *T. gondii* RH, and after ~20 divisions they begin to differentiate into slower growing parasites that express bradyzoite markers, suggesting that there may be a developmental clock controlling this spontaneous conversion (Jerome et al., 1998). Taken together, these findings suggest that there is some type of parasite intrinsic factor that enables these parasites to transition to bradyzoites following initial tachyzoite expansion.

Host factors, specifically the differentiation state of a given host cell, can also impact *T. gondii* cystogenesis. *T. gondii* forms tissue cysts when grown in mouse primary skeletal muscle cells that have been differentiated into polynucleated myotubes, which are withdrawn from cell cycle progression (Ferreira-da-Silva et al., 2009; Swierzy and Lüder, 2015). When these host cells are genetically manipulated to knock-down Testis-specific Y-encoded-like protein 2 (Tspyl2), a negative cell cycle regulator that contributes withdraw from host cell cycle progression in these myotubes, *T. gondii* fails to form tissue cysts in these cells (Swierzy and Lüder, 2015). Additionally, expression levels of the host gene human cell division autoantigen-1 (CDA1) are important for bradyzoite development. *T. gondii* has been shown to grow slower and express bradyzoite specific genes when grown in host cells treated (pre-treatment or continuous treatment) with the trisubstituted pyrrole small molecule Compound 1, which upregulates expression of host CDA1 (Radke et al., 2006). CDA1 is a negative regulator of cell growth and has regions with homology to testis protein TSPY (Chai et al., 2001). Additionally, *T. gondii* forms tissue cysts when grown in HeLa cells that overexpress a transgene encoding CDA1 (Radke et al., 2006). The ability of cells that have withdrawn from their cell cycle to promote tissue cyst formation in *T. gondii* is a likely contributor to the preference *T. gondii* shows for muscular tissue and the tissues of the central nervous system.

Several well characterized stressors are known to induce *T. gondii* tachyzoite-to-bradyzoite stage conversion in vitro (summarized in **Table 1**). Perhaps the most well-known stressor that leads to robust formation of *T. gondii* tissue cysts in vitro is alkaline pH (pH ~8 as compared to the standard pH growth conditions of 7.2–7.4). Multiple groups have shown that alkaline pH induces *T. gondii* cyst development either when applied to host cells after infection (Soête et al., 1994) or when applied to extracellular parasites prior to infection (Weiss et al., 1998). Despite robust induction of *T. gondii* tissue cyst development, the exact mechanism remains unknown. It is possible that alkaline pH derived stress induces a myriad of both host and parasite derived signals that are needed in order to initiate bradyzoite development in *T. gondii*. Furthermore, treatment of infected host cells with pH 6.8 media has also been shown to induce bradyzoite development (Weiss et al., 1995). In addition to changes in pH, heat shock (43°C as opposed to 37°C) and sodium arsenite treatment of infected host cells have also been shown to induce stage conversion in *T. gondii*, however heat shock is not an optimal method for inducing bradyzoite development as it often results in decreased parasite invasion, parasite killing, and loss of host cell viability (Soête et al., 1994). Nutrient starvation can also induce stage conversion in *T. gondii*. Pyrimidine starvation achieved via deletion of the uracil phosphoribosyl transferase (UPRT) gene in combination with growth in atmospheric CO_2_ (0.03% compared to 5%) also leads to a parasite growth reduction and expression of bradyzoite markers (Bohne and Roos, 1997). Arginine starvation also decreases the replication of *T. gondii* and induces tissue cyst formation (Fox et al., 2004). Cholesterol depletion via growth in media supplemented with lipoprotein depleted serum (as compared to growth in 5% fetal bovine serum) has also been shown to induce bradyzoite gene expression (Ihara and Nishikawa, 2014). Additionally, interferon gamma (IFN-γ) treatment of *T. gondii* infected macrophages results in expression of bradyzoite specific antigens (Bohne et al., 1994), however IFN-γ is not capable of inducing expression of bradyzoite specific antigens in human fibroblasts (Suzuki et al., 1989; Bohne et al., 1993; Soête et al., 1994; Weiss et al., 1995). Other in vitro stressors that may mimic IFN-γ-driven immune pressure also induces *T. gondii* cystogenesis, such as the production of nitric oxide. Exogeneous nitric oxide produced from sodium nitroprusside (SNP) treatment can induce bradyzoite development (Bohne et al., 1994). Exogenous nitric oxide likely inhibits proteins involved in the electron transport chain, thus treatment with mitochondrial inhibitors oligomycin, antimycin A, (Bohne et al., 1994), and atovaquone (Tomavo and Boothroyd, 1995) can also induce expression of bradyzoite antigens in *T. gondii*. Overall, the numerous and diverse exogenous stressors capable of inducing bradyzoite development in *T. gondii* suggest that multiple signals can be used as triggers to induce the fundamental process of bradyzoite formation.

**Table 1:** Summary of exogenous stressors that induce bradyzoite development in *Toxoplasma gondii*.

Stressor	Parasite life stage used for infection	Parasite strain	Host cell	Method used to determine bradyzoite formation	Citation
Alkaline pH (treatment of infected host cells)	Sporozoites	VEG	Human foreskin fibroblasts (HFFs)	Bradyzoite-specific antibodies	(Jerome et al., 1998)
	*In vivo* tachyzoites	RH	HFFs; Vero cells	Bradyzoite-specific antibodies	(Soête et al., 1994)
Alkaline pH (extracellular parasites)	*In vitro* tachyzoites	ME49	HFFs	Bradyzoite-specific antibodies	(Weiss et al., 1998)
Heat Shock (43 degrees C)	*In vivo* tachyzoites	RH	Vero cells	Bradyzoite-specific antibodies	(Soête et al., 1994)
Sodium arsenite	*In vivo* tachyzoites	RH	Vero cells	Bradyzoite-specific antibodies	(Soête et al., 1994)
Sodium nitroprusside (SNP) (extracellular parasites)	*In vitro* tachyzoites	ME49	HFF	Bradyzoite-specific antibodies	(Weiss et al., 1998)
SNP (infected host cells)	*In vitro* tachyzoites	NTE	Murine bone marrow-derived macrophages (BMDM)	Bradyzoite-specific antibodies	(Bohne et al., 1994)
Interferon gamma	*In vitro* derived tachyzoites	NTE	Murine peritoneal macrophages	Bradyzoite-specific antibodies	(Bohne et al., 1993)
	*In vitr*o tachyzoites	NTE	Murine BMDM	Bradyzoite-specific antibodies	(Bohne et al., 1994)
Antimycin A	*In vitro* tachyzoites	NTE	Murine BMDM	Bradyzoite-specific antibodies	(Bohne et al., 1994)
Oligomycin	*In vitro* tachyzoites	NTE	Human fibroblasts	Bradyzoite-specific antibodies	(Bohne et al., 1994)
Atovaquone	*In vitro* tachyzoites	PLK	HFFs	Bradyzoite-specific antibodies	(Tomavo and Boothroyd, 1995)
Arginine starvation	*In vitro* tachyzoites	RH; PLK	HFFs	Dolichos biflorus agglutinin	(Fox et al., 2004)
Pyrimidine starvation	*In vitro* tachyzoites	RHδUPRT	HFFs	Bradyzoite-specific antibodies	(Bohne and Roos, 1997)
Cholesterol depletion (Lipoprotein depleted serum)	*In vitro* tachyzoites	ME49	Chinese hamster ovary cells	Bradyzoite-specific antibodies	(Ihara and Nishikawa, 2014)
Compound 1	*In vitro* tachyzoites	ME49B7; Pru; VEG; CTG	HFFs	Bradyzoite-specific antibodies; Dolichos biflorus agglutinin	(Radke et al., 2006)

In comparison to in vitro systems, in vivo factors that induce cystogenesis are much less clear. Tumor necrosis factor-alpha (TNF-α) and inducible nitric oxide synthase (iNOS) may play a role in restricting cystogenesis, as TNF receptor p55- and p75-deficient mice and iNOS deficient mice develop more tissue cysts in the brain compared to wild type mice despite relatively equivalent parasite burden in peritoneal cells early in infection. Interestingly, mice deficient for TNF-α receptors or iNOS succumb to chronic infection while the WT parasites survive (Scharton-Kersten et al., 1997; Yap et al., 1998). It is challenging to determine if the increase in tissue cysts in the brains of these mice is a result of TNF-α and iNOS restricting tissue cyst development, or if more parasites make it to the brain prior to tissue cyst formation. Future experiments using live imaging that quantifies parasite burden could be useful to test these hypotheses. Additionally, CD4+ and CD8+ T-cells are important for the maintenance of chronic infection characterized by the bradyzoite/tissue cyst life stage. Depletion of these cells with neutralizing antibodies leads to reactivation of *T. gondii* infection leading to parasite proliferation (Gazzinelli et al., 1992). It is also thought that IFN-γ may play a critical role in cystogenesis. It is hypothesized that IFN-γ contributes to the initiation of tissue cyst formation in vivo, as it can induce cystogenesis in vitro (described above), however IFN-γ knockout mice fail to control acute proliferation of parasites and succumb to acute infection (Suzuki et al., 1988; Suzuki et al., 1989) even when infected with avirulent strains of *T. gondii* (Coombs et al., 2020) making it difficult to determine if IFN-γ induces cystogenesis in vivo. It is also possible that *T. gondii* spontaneously forms tissue cysts during in vivo infections, however this spontaneous development is again challenging to observe experimentally due to the lethality of *T. gondii* infections in mice with disrupted immune systems. Linking what is known about in vitro cyst development in *T. gondii* to what happens in vivo is a significant, but important, knowledge gap in the field that will require new technological innovation to fill.

### Hammondia hammondi

In comparison to *T. gondii*, little is known about bradyzoite and tissue cyst formation in *H. hammondi*. When grown in vitro, *H. hammondi* fails to grow in continuous culture and spontaneously undergoes a terminal differentiation process where it completely converts to tissue cysts that are only infectious to definitive feline hosts (Sheffield et al., 1976; Riahi et al., 1995; Dubey and Sreekumar, 2003; Sokol et al., 2018). The timing of tissue cyst formation corresponds to when *H. hammondi* parasites lose their ability to infect a new host cell (Sokol et al., 2018), demonstrating that *H. hammondi* follows a strict obligate heteroxenous life cycle even in in vitro growth conditions (a sharp contrast to both *T. gondii* and *N. caninum)*. Furthermore, comparative transcriptomic analysis between replicating *T. gondii* and *H. hammondi* showed that the *H. hammondi* transcriptional profile is enriched for genes that are typically reserved for expression during bradyzoite and sexual stages occurring in feline intestinal cells in *T. gondii* (Sokol et al., 2018). It is likely that *H. hammondi* follows a strictly regulated life cycle where it is poised to convert to its next life stages after a pre-defined time as a given life form.

**Figure 2:**
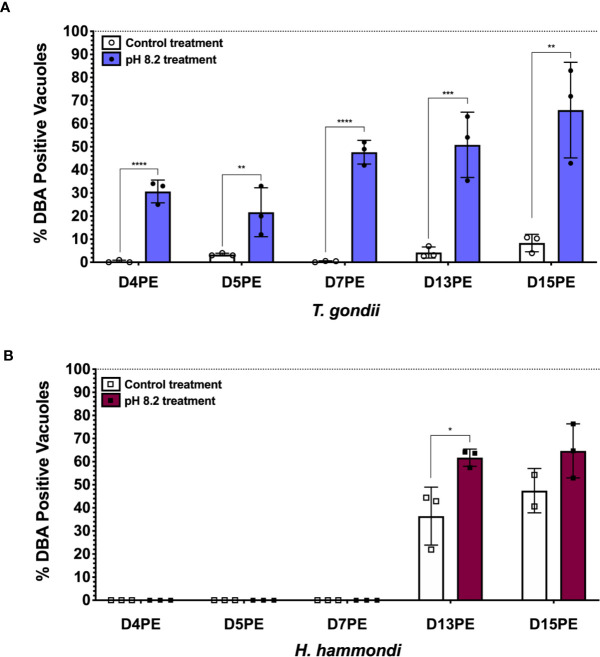
*H. hammondi* can respond to stress conditions when exposed to alkaline pH 13 days post excystation (DPE) from oocysts. **(A)** Percentage of DBA positive vacuoles observed following 48 h of alkaline pH stress initially applied at D4, 5, 7, 13, and 15PE for *T. gondii* VEG. **(B)** Percentage of DBA positive vacuoles observed following 48 h of alkaline pH stress initially applied at D4, 5, 7, 13, and 15PE for *H. hammondi* American. Statistical significance was determined by 2way ANOVA with Sidak’s multiple comparisons test of arcsine transformed data. (N = 2–3 biological replicates, *P = 0.03, **P < 0.01, ***P < 0.001, and ****P < 0.0001) This experiment, with the exception of time of alkaline stress application at the time points mentioned above, was performed as previously described (Sokol et al., 2018).

Even though *H. hammondi* completely and spontaneously forms tissue cysts when grown in vitro, it cannot be induced to form tissue cysts with alkaline pH, a robust inducer of stage conversion in *T. gondii*, at early time points following sporozoite-initiated infection (Sokol et al., 2018). This discovery was important because it suggested that the ability to constitutively respond to alkaline pH in *T. gondii* was a derived trait. Since this initial work we have also investigated if *H. hammondi* is eventually able to form tissue cysts in response to alkaline pH derived stress applied at later stages in its in vitro cycle. Interestingly, we have found that when alkaline pH derived stress is applied for 48 h at later developmental time points (Day 13 post sporozoite-derived infection), significantly more tissue cysts form in response to alkaline pH treatment than occur spontaneously at this time (**Figure 2**). This finding suggests that as *H. hammondi* progresses through a predefined developmental program it differentiates from a life form that is incapable of responding to alkaline pH (unable to sense and/or respond) to a life form that is capable of sensing and/or responding. This hints at a conserved linkage between alkaline pH responsiveness and the tachyzoite to bradyzoite stage conversion. Future work investigating the differences in gene expression between these time points could be promising in uncovering additional components of the mechanisms that these parasites use to sense and respond to their environment. It is currently unknown as to how *H. hammondi* responds to other stressors known to induce tissue cyst development in *T. gondii*.

### Neospora caninum

Like *T. gondii*, *N. caninum* alternates between two life stages presumably to survive host immune responses. The mechanisms underlying *N. caninum* tachyzoite to bradyzoite conversion remain largely unknown (to an even greater extent than for *H. hammondi*) due to difficulties in developing in vitro models for bradyzoite development. Methods used to obtain *T. gondii* bradyzoites in vitro are ineffective or inefficient for *N. caninum* cyst formation. Although nitric oxide treatment of murine keratinocytes infected with *N. caninum* tachyzoites yields cysts, they are surrounded by thick keratin filament bundles and thus impede parasite purification processes (Vonlaufen et al., 2002). Very few studies have investigated the effects of stress on stage conversion for *N. caninum*. One study suggests that nitric oxide, increasing pH, or increasing temperature can increase tachyzoite to bradyzoite conversion in *N. caninum* (Weiss et al., 1999). SNP has also been shown to increase expression of *N. caninum* bradyzoite and cyst wall markers (Risco-Castillo et al., 2004). However, there are no studies investigating any difference in gene expression during the tachyzoite to bradyzoite conversion process in *N. caninum*. Substantial work with regards to how to identify *N. caninum* bradyzoites and reliably produce this life stage in vitro and in vivo is still needed in order to expand our understanding of how stage conversion occurs in *N. caninum*.

## Parasite Intrinsic Molecular Mechanisms of Stage Conversion

### Chromatin

**Figure 3:**
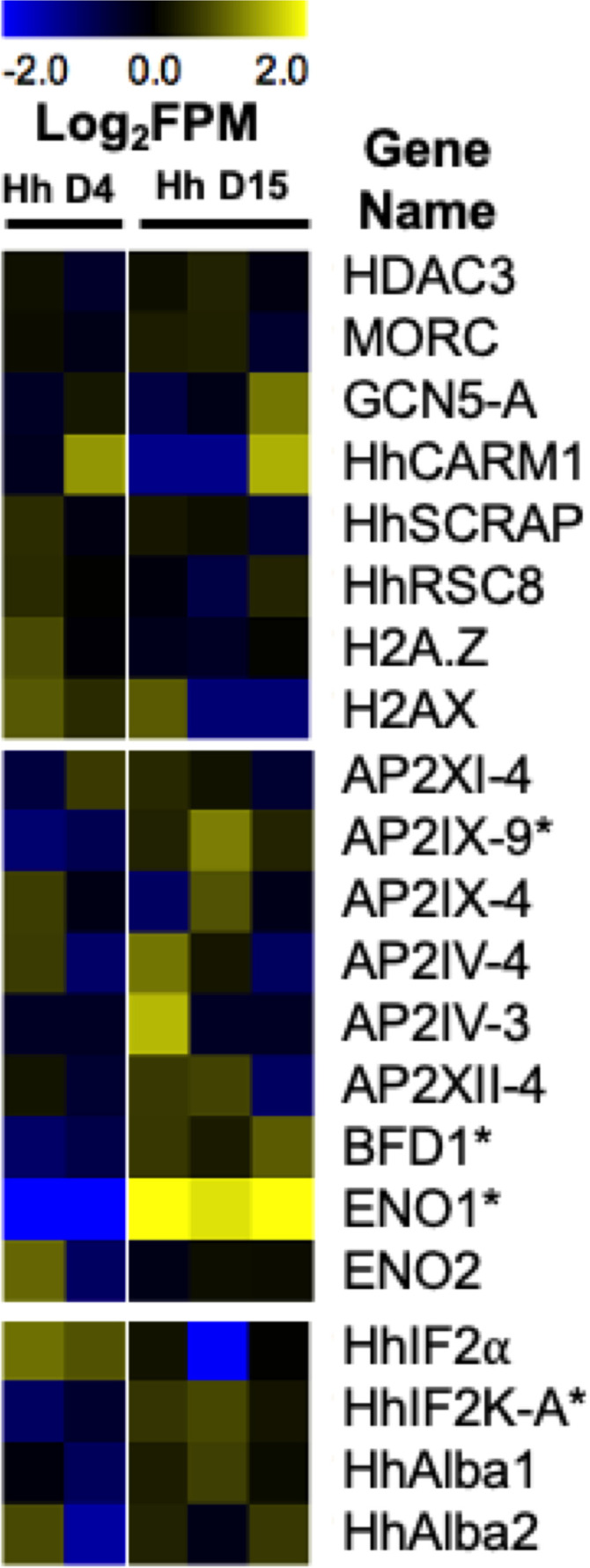
Heatmap representing *H. hammondi* transcriptional abundance during spontaneous development of known regulators of bradyzoite formation. Heatmaps show mean centered Log_2_FPM values. Asterisk (*) represent genes with significant differences between D4 (N = 2 biological replicates) and D15 (N = 3 biological replicates) samples. Significance is defined as |Log_2_ Fold Change| >1 and P_adj_ < 0.01. *T. gondii* gene IDs for these genes and the gene IDs for each orthologs in *H. hammondi* and *N. caninum* are found in **Table 2**. The *H. hammond*i transcriptional data used to generate this figure was obtained from (Sokol et al., 2018).

**Figure 4:**
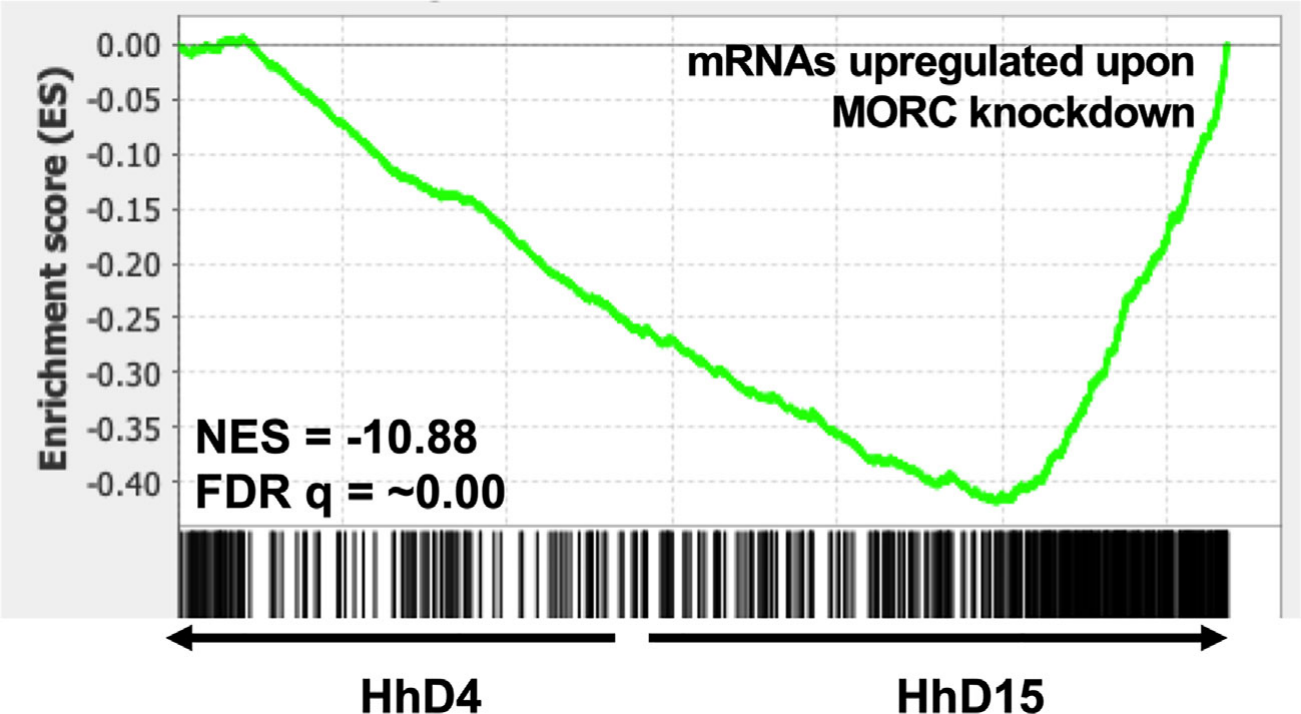
Spontaneously developing *H. hammondi* transcriptional abundance is enriched for genes regulated by MORC. Preranked GSEA, comparing D4 and D15 HhEth1, ranked listed was calculated from Log_2_ fold change between D4 and D15 spontaneous developing HhEth1 samples. The data used to generate this figure was obtained from (Sokol et al., 2018). Gene sets were created with data from (Farhat et al., 2020).

Stage conversion in eukaryotic parasites is accompanied by significant changes in patterns of gene expression (Gomez et al., 2010; Chen et al., 2018). The chromatin landscape of a given life stage plays a large role in what genes are expressed, therefore playing a critical role in stage conversion. Several chromatin remodeling factors have been identified in *T. gondii* that play a role in altering the chromatin landscape during different life stages. One such factor is Histone Deacetylase 3 (HDAC3), which is a component of *T. gondii’s* corepressor complex. HDAC3 is associated with bradyzoite specific promoters (Saksouk et al., 2005) and when its activity is inhibited with the compound FR235222, expression of bradyzoite genes increases (Bougdour et al., 2009). These findings suggest that histone deacetylation via HDAC3 functions to repress the expression of bradyzoite genes and keep *T. gondii* in a tachyzoite life form. HDAC3 has been shown to work with the recently discovered *T. gondii* microrchidia (MORC) protein. MORC interacts with several AP2 transcription factors (Farhat et al., 2020) [including AP2IX-4 and AP2XII-2 discussed below (Srivastava et al., 2020)] and recruits HDAC3 to chromatin, enabling the generation of hypo-acetylated chromatin which represses gene expression. When MORC is depleted in *T. gondii*, the parasites begin to express transcripts typically expressed in other life stages, such as merozoite and oocysts specific genes, that are restricted to the *T. gondii* sexual stages (Farhat et al., 2020). These findings suggest that MORC functions as a repressor of sexual development associated gene expression through chromatin modification in *T. gondii*. Our transcriptional data from replicating *H. hammondi* on Day 4 (D4) and Day 15 (D15) post sporozoite infection, does not show any changes in transcriptional abundance of either of *H. hammondi’s* orthologs of HDAC3 or MORC (**Figure 3**). However, in comparison to *T. gondii*, *H. hammondi’s* transcriptional profile is enriched for genes that are associated with sexual development in *T. gondii* (Sokol et al., 2018), a process which has been shown to be repressed by MORC (Farhat et al., 2020). Because of this similarity, we hypothesized that transcript abundance of MORC repressed genes would be enriched in *H. hammondi* at D15 compared to D4. To test this hypothesis, we conducted pre-ranked gene set enrichment analysis (GSEA) as previously described (Subramanian et al., 2005) and found significant enrichment for MORC repressed transcripts [gene sets derived from data published in (Farhat et al., 2020)] (NES= -10.88, FDRq = ~0.00 ) in D15 *H. hammondi* (**Figure 4**). This analysis suggests that transcriptional changes in spontaneously developing *H. hammondi* resemble some of the transcriptional changes observed in MORC depleted *T. gondii*. It would be interesting to further investigate if altering MORC levels could alter *H. hammondi’s* terminal differentiation phenotype.

Another histone remodeling enzyme found in *T. gondii* is GCN5-A, a lysine histone acetyltransferase. This enzyme plays an oppositional role to HDAC3. GCN5-A is found at tachyzoite promoters when *T. gondii* is grown under tachyzoite growth conditions (Saksouk et al., 2005). Additionally, ChIP qPCR experiments have shown that when *T. gondii* is exposed to alkaline pH stress conditions, GCN5-A occupancy is enriched in the promotors of bradyzoite specific genes that are upregulated in response to stress. When GCN5-A is knocked out in *T. gondii*, the parasites are unable to upregulate 74% of known bradyzoite genes in response to alkaline pH induced stress (Naguleswaran et al., 2010). Together, these findings demonstrate that GCN5-A plays a critical role in *T. gondii’s* ability to alter its gene expression in response to alkaline pH stress.

Additional chromatin remodeling enzymes that play a role in the alteration of stage conversion associated gene expression have also been identified in *T. gondii*. These include TgCARM1 (Saksouk et al., 2005), TgSCRAP (Sullivan et al., 2003), and TgRSC8 (Craver et al., 2010; Rooney et al., 2011). TgCARM1 is a histone arginine methyltransferase protein that is essential for parasite replication. When N-methyltransferase activity is inhibited with the small molecule AMI-1 via pretreatment of extracellular parasites, *T. gondii* forms significantly more bradyzoites compared to a vehicle control (Saksouk et al., 2005). TgSCRAP, a Snf2-related CBP activator protein and SNF/SWI chromatin remodeler, upregulates the expression of the known bradyzoite gene bradyzoite antigen 1 (BAG1) during alkaline pH induced stress, and has been shown to enhance CREB (cAMP response element binding protein) mediated transcription, which may suggest a role in the protein kinase A signaling pathway that has also been implicated in bradyzoite development for *T. gondii* (Sullivan et al., 2003). Another chromatin remodeling complex that contributes to bradyzoite development is TgRSC8, a homolog of the nucleosome remodeling complex Rsc8p protein in *Saccharomyces cerevisiae*. When this protein is mutated in *T. gondii*, some bradyzoite genes showed a significant reduction in transcript abundance when exposed to alkaline pH stress conditions. However, these mutants did not show reduced Dolichos biflorus agglutinin (DBA) staining (Rooney et al., 2011), which specifically recognizes the glycosylated cyst wall protein CST1 (Zhang et al., 2001). In addition to chromatin remodelers, histone variants have also been implicated in stage conversion in *T. gondii*. The histone variant H2A.Z is expressed in mature, in vivo bradyzoites and H2AX is also expressed in mature, in vivo bradyzoites but also displays increased expression in vitro during alkaline pH stress (Dalmasso et al., 2009). All together, these findings suggest that the alteration of chromatin is an important contributor to stage conversion associated gene expression in *T. gondii*.

Despite having orthologs of all of these chromatin remodeling enzymes and histone variants, our understanding of their role in bradyzoite formation in *H. hammondi* and *N. caninum* is mostly unclear. Our transcriptional data from replicating *H. hammondi* show no significant changes in the transcript abundance for any of the *H. hammondi* orthologs during spontaneous development (**Figure 3**). However, these findings could suggest that transcriptional abundance of these genes is needed similarly in all life stages and that the mechanisms controlling their translation, activation, or their recruitment to specific genes are important for initiating the tachyzoite to bradyzoite developmental transitions.

### Transcription Factors

Transcription factors are an important class of proteins that play a significant role in the control of stage conversion specific gene expression and have long been investigated for their role in stage conversion associated gene expression in *T. gondii*. One of the first major classes of transcription factors investigated for their role in stage conversion in *T. gondii* were the AP2 transcription factors. AP2 factors were first identified in Apicomplexans in 2005. These transcription factors share the Apetala2 (AP2) integrase DNA binding domain typically found in transcription factors of numerous plant species (Balaji et al., 2005). AP2 transcription factors are known to play a major role in stage conversion in *Plasmodium* species (Painter et al., 2011). There are currently 67 AP2 factors (66 annotated as AP2 domain transcription factors and 1 annotated as an AP2 domain-containing protein) in the *T. gondii* ME49 genome (Gajria et al., 2008). However, only 6 of these AP2 transcription factors have been tied to bradyzoite development in *T. gondii*. The AP2 transcription factor AP2IX-9 functions mainly as a repressor of bradyzoite development, maintaining parasites in an intermediate, pre-bradyzoite state. AP2IX-9 binds to the CAGTGT motif and functions to repress transcription (Radke et al., 2013). Furthermore, deletion of AP2IX-9 results in increased tissue cyst formation in parasites cultivated in normal growth conditions (Hong et al., 2017). During spontaneous development (D4 versus D15) in *H. hammondi*, we see significant increases in transcriptional abundance of the *H. hammondi* ortholog of AP2IX-9 (|Log_2_ Fold Change| = 2.21, P_adj_ = 0.009). Since AP2IX-9 is known to keep parasites in a pre-bradyzoite state, this data could indicate that D15 *H. hammondi* are being maintained in a pre-bradyzoite like state, prior to their complete terminal differentiation which is first observed at D23 post sporozoite derived infection (Sokol et al., 2018). Another AP2 factor, AP2XII-2, has been shown to interact with the MORC protein and results in increased tissue cyst formation in vitro upon knockdown in *T. gondii*, suggesting that this factor may be important for maintaining tachyzoites (Srivastava et al., 2020). Additionally, AP2 transcription factors have been identified in *T. gondii* that are involved in promoting bradyzoite development. These include AP2IV-4 (Radke et al., 2018), AP2IV-3 (Hong et al., 2017), AP2IX-4 (Huang et al., 2017), and AP2XI-4 (Walker et al., 2013). When these AP2 transcription factors are deleted, parasites have a decreased ability to form tissue cysts in vitro and/or in vivo. Furthermore, alteration (either deletion or overexpression) of these factors results in significant differences in transcript abundance of known bradyzoite genes (Walker et al., 2013; Hong et al., 2017; Huang et al., 2017; Radke et al., 2018). Our *H. hammondi* transcriptional data does not show any significant differences in transcript abundance in these AP2 factors during spontaneous development in *H. hammondi* (**Figure 3**).

In addition to AP2 transcription factors, *T. gondii* has additional transcription factors that are critical for stage conversion. One such transcription factor is Bradyzoite Formation Deficient 1 (BFD1). BFD1 was recently identified as a master transcriptional regulator of bradyzoite development in *T. gondii* using a large-scale genetic screen. BFD1 is a nuclear localized Myb-like DNA binding protein that binds to the CACTGG motif near that transcriptional start site of differentially regulated genes. When BFD1 is deleted in *T. gondii*, these parasites fail to form tissue cysts in vitro in response to alkaline pH derived stress and treatment with Compound 1. These knockout parasites also fail to form tissue cysts in vivo. Furthermore, BFD1 knockout parasites fail to express several bradyzoite specific genes when treated with alkaline pH, suggesting that BFD1 plays a major role in initiating stage conversion associated gene expression (Waldman et al., 2020). Transcriptional abundance of the *H. hammondi* ortholog of BFD1 is modestly but significantly upregulated (|Log_2_ Fold Change| = 1.47, P_adj_ = <0.001) during spontaneous development (**Figure 3**), suggesting that increases in BFD1 expression in *H. hammondi* may play an important role in *H. hammondi’s* terminal differentiation phenotype.

Additional *T. gondii* proteins that may play a role in stage conversion are the glycolytic enzymes, Enolase1 (ENO1) and Enolase 2 (ENO2). ENO1 is specifically expressed in bradyzoites and is a known bradyzoite specific gene. ENO2 is expressed in tachyzoites and can be found localized in the cytoplasm and nucleus (Ferguson et al., 2002). When ENO1 is deleted in *T. gondii*, the ability of the parasite to form in vivo cysts is impaired. Both ENO1 and ENO2 have been shown to bind promoters in ChIPseq and ChIP qPCR experiments and ENO1 has been shown to bind the ENO2 promoter. Furthermore, analysis of the ENO1 promoter revealed a stress response element that could be activated with nuclear extracts purified from bradyzoites (Kibe et al., 2005). Together, this data demonstrates a role as transcription factors implicated in stage conversion for ENO1 and ENO2. ENO1 is significantly upregulated during spontaneous development in *H. hammondi* (**Figure 3**), however it is possible that this increase could also be due to ENO1’s metabolic role for bradyzoites.

### RNA-Binding Proteins

While stage conversion is typically accompanied by global changes in transcript abundance in Apicomplexan parasites, translation control is an additional component needed for stage conversion associated gene expression. Several RNA binding proteins have been identified in *T. gondii* and some have been implicated in the conversion between tachyzoites and bradyzoites. Such factors include TgIF2α/eIF2 (eukaryotic initiation factor 2 alpha) and TgIF2K-A (initiation factor 2 kinase -A). TgIF2K-A is responsible for phosphorylating and activating TgIF2α, which functions as an inhibitor of translation initiation. TgIF2α is phosphorylated in response to stress derived from both alkaline pH and heat shock (Sullivan et al., 2004). TgIF2α phosphorylation is maintained in bradyzoites. When dephosphorylation of TgIF2α is inhibited, parasites both increase the transcriptional abundance of known bradyzoites genes and form DBA positive tissue cysts (Narasimhan et al., 2008). Furthermore, when the activity of TgIF2K-A is inhibited in *T. gondii*, thus preventing TgIF2α phosphorylation, these parasites form significantly fewer tissue cysts when exposed to alkaline pH stress (Augusto et al., 2018). Our transcriptional data from replicating *H. hammondi* shows that transcript abundance of HhIF2K-A is significantly upregulated during spontaneous development ((|Log_2_ Fold Change| = 1.22, P_adj_ = <0.001) (**Figure 3**) (Sokol et al., 2018). This increase in transcriptional abundance could correlate to increased IF2α phosphorylation and translational inhibition in *H. hammondi*. Additional experiments investigating HhIF2α phosphorylation and HhIF2K-A activity during bradyzoite formation in *H. hammondi* would be helpful in identifying translation control as a conserved mechanism of stage conversion.

Additionally, *T. gondii* encodes two RNA binding proteins related to Alba proteins in archaea, TgAlba1 and TgAlba2. In response to alkaline pH derived stress, both TgAlba1 and TgAlba2 colocalize with RNA granules. These proteins bind greater than 30 RNAs, including their own. TgAlba1 binds to the promoter of TgAlba2. Upon deletion of TgAlba1, TgAlba2 is no longer translated. Furthermore, deletion of TgAlba1 in *T. gondii* also leads to decreased tissue cyst formation in vitro (in response to alkaline pH stress) and in vivo (Gissot et al., 2013). However, we do not see significant changes in transcriptional abundance in the *H. hammondi* orthologs of TgAlba1 and TgAlba2 (**Figure 3**). Together, these examples of RNA binding proteins found in *T. gondii* demonstrate that translational control plays a critical role in stage conversion associated gene expression.

## Conclusions

The tachyzoite to bradyzoite transition is a fundamental developmental process for the success of *T. gondii* and its closest relatives *H. hammondi* and *N. caninum*, as it is necessary for the survival and transmission of these parasite species. Some similarities in exogenous stressors that induce bradyzoite formation exist between species, indicating that there is some conservation in the way that these parasites sense stress. Moreover, the genetic components involved in the mechanisms governing stage conversion between tachyzoite and bradyzoite life stages are only starting to be uncovered for *T. gondii*, but so far include several factors that are needed for both transcriptional and translational control. Both *H. hammondi* and *N. caninum* have syntenic orthologs of all of these genetic components identified in *T. gondii* (summarized in **Table 2**), suggesting that they may also play a role in stage conversion in these species as well. However, only a few of these genes have altered transcriptional abundance in spontaneously differentiating *H. hammondi*. These observations from *H. hammondi* suggest that the mechanisms controlling stage conversion may function differently in *H. hammondi* and may rely on more than transcriptional regulation alone in order to induce bradyzoite development, which is not surprising given the importance of the tachyzoite to bradyzoite transition and the complexity of stage conversion associated gene expression. Together, these studies indicate that future work aimed at linking these factors in gene regulatory networks, in addition to identifying new factors, is needed to contribute to a better understanding of how these parasites initiate the global changes in stage conversion associated gene expression on a mechanistic level.

**Table 2:** Summary of genes known to play a role in stage conversion in *T. gondii*.

Gene Name	Role in stage conversion associated gene expression	Gene ID *T. gondii (from Toxodb.org)*	Gene ID *H. hammondi* ortholog*(from Toxodb.org)*	Gene ID *N. caninum* ortholog*(from Toxodb.org)*
HDAC3	Represses transcription	TgME49_227290	HHA_227290	NCLIV_045860
MORC	Works with HDA3 and AP2 factors to repress transcription	TgME49_305340	HHA_305340	NCLIV_043930
GCN5-A	Activates transcriptions	TgME49_254555	HHA_254555	NCLIV_008840
CARM1	Activity needed to maintain tachyzoites	TgME49_294270	HHA_294270	NCLIV_001020
SCRAP	Upregulates BAG1 expression	TgME49_280800	HHA_280800	NCLIV_019390
RSC8	Regulates transcription of bradyzoite genes	TgME49_286920	HHA_286920	NCLIV_013840
H2A.Z	Expressed in bradyzoites; function in stage conversion is unknown	TgME49_300200	HHA_300200	NCLIV_064530
H2AX	Expressed in bradyzoites; function in stage conversion is unknown	TgME49_261580	HHA_261580	NCLIV_025910
AP2XI-4	Promotes bradyzoite formation	TGME49_315760	HHA_315760	NCLIV_058430
AP2IX-9	Maintains pre-bradyzoites	TgME49_306620	HHA_306620	NCLIV_044800
AP2IX-4	Promotes bradyzoite formation	TgME49_288950	HHA_288950	NCLIV_041340
AP2IV-4	Promotes bradyzoite formation	TgME49_318470	HHA_318470	NCLIV_011080
AP2IV-3	Promotes bradyzoite formation	TgME49_318610	HHA_318610	NCLIV_010930
AP2XII-4	Interacts with MORC, maintains tachyzoites	TgME49_217700	HHA_217700	NCLIV_062490
BFD1	Required for bradyzoite formation	TgME49_200385	HHA_200385	NCLIV_038230
ENO1	Promotes bradyzoite formation	TgME49_268860	HHA_268860	NCLIV_037490
ENO2	Expressed in tachyzoite nuclei, function in stage conversion is unknown	TgME49_268850	HHA_268850	NCLIV_037500
IF2a	Phosphorylated in bradyzoites, leads to increase in bradyzoite specific gene expression	TgME49_258740	HHA_258740	NCLIV_027770
IF2K-A	Phosphorylates IF2a, activity promotes bradyzoite formation	TgME49_229630	HHA_229630	NCLIV_030460
Alba1	Promotes bradyzoite formation	TgME49_221380	HHA_221380	NCLIV_004920
Alba2	Promotes bradyzoite formation	TgME49_218820	HHA_218820	NCLIV_061560

## Author Contributions

SS-B, RC, and JB conceptualized, wrote, and edited this manuscript. All authors contributed to the article and approved the submitted version.

## Funding

This work was supported by grants F31AI140529 to SS-B and R01AI116855 to JB.

## Conflict of Interest

The authors declare that the research was conducted in the absence of any commercial or financial relationships that could be construed as a potential conflict of interest.
